# Closed MCP‐Mod for Pairwise Comparisons of Several Doses With a Control

**DOI:** 10.1002/sim.70124

**Published:** 2025-05-19

**Authors:** Franz Koenig, Sergey Krasnozhon, Bjoern Bornkamp, Frank Bretz, Ekkehard Glimm, Alexandra Graf, Dong Xi

**Affiliations:** ^1^ Center for Medical Data Science Medical University of Vienna Vienna Austria; ^2^ Novartis Pharma AG Basel Switzerland; ^3^ Medical Faculty Otto‐von‐Guericke‐University of Magdeburg Magdeburg Germany; ^4^ Gilead Sciences Foster City California USA

**Keywords:** contrast tests, dose finding, dose–response, many‐to‐one comparisons, modeling

## Abstract

The MCP‐Mod approach by Bretz et al. is commonly applied for dose–response testing and estimation in clinical trials. The MCP part of MCP‐Mod was originally developed to detect a dose–response signal using a multiple contrast test, but it is not appropriate to make a specific claim that the drug has a positive effect at an individual dose. In this paper, we extend the MCP‐Mod approach to obtain confirmatory *p*‐values for detecting a dose–response signal as well as for the pairwise comparisons of the individual doses against placebo. We apply the closed test principle from Marcus et al. to the optimal contrast tests based on a candidate set of plausible dose–response shapes available at the planning stage of a clinical trial. We show that the contrast coefficients have to be optimized under suitable constraints to guarantee strong Type 1 error rate control at a pre‐specified significance level. Motivated by a recent clinical trial, we evaluate the operating characteristics of the proposed methods in a comprehensive simulation study.

## 1 Introduction

Knowledge of the relationships among dose, drug‐concentration in blood, and clinical response (effectiveness and undesirable effects) is important for the safe and effective use of drugs in individual patients. This information can help identify an appropriate starting dose, the best way to adjust dosage to the needs of a particular patient, and a dose beyond which increases would be unlikely to provide added benefit or would produce unacceptable side effects [1]. Comparing multiple doses against a common control in trials relevant for market authorization (e.g., pivotal Phase III trials) results in a multiple hypothesis test problem. Regulatory guidelines for drug development then suggest a strong control of the familywise error rate (FWER), that is, the probability to erroneously reject at least one true null hypothesis should be bounded by a pre‐specified significance level $\alpha \in \left(0,1\right)$ [2, 3, 4].

Standard FWER‐controlling multiple comparison procedures (MCP) to evaluate the pairwise comparisons between several doses of a new treatment and placebo include the Dunnett test [5] and its stepwise extensions [6, 7] as well as the hierarchical test [8]. However, such approaches become less attractive if the number of doses is relatively large, as one would intuitively expect to be able to borrow information across doses. This has led to the development of hybrid approaches, such as the MCP‐Mod approach [9], that combine basic principles of multiple comparison procedures with modeling (Mod) techniques; see also [10] for an early reference. The key idea is to specify at the trial design stage a candidate set of plausible dose–response models, in discussion with the clinical team, based on available pharmacologic information, dose–response information from similar compounds, data from earlier trials, etc. This candidate set then gives rise to a set of optimal contrasts used to test for the presence of a dose–response signal, where the contrast coefficients are chosen as to maximize the probability of detecting particular dose–response alternatives (i.e., the candidate set of dose–response shapes). If any of the contrast tests is statistically significant, one concludes in favor of a significant dose–response signal and moves on with a suitable model selection or model averaging approach for dose–response and target dose estimation.

Since its original publication, MCP‐Mod has been subject to several investigations [11]. discussed practical considerations regarding the implementation of this methodology, including sample size calculations for the MCP part. Likewise [12], provided further recommendations when implementing MCP‐Mod, including general considerations on when to apply MCP‐Mod as well as specific considerations applicable at the trial design and analysis stages [13]. constructed optimal designs for the estimation of target doses to determine the optimum location of the dose levels within the dose range under investigation and allocation of patients to the individual dose levels, taking model uncertainty into account [14]. described the use of the MCP‐Mod approach for general parametric models (e.g., for time‐to‐event endpoints and including longitudinal data modeling), see also [15, 16] for specific extensions of the MCP‐Mod approach to binary data. Several authors investigated the use of likelihood ratio tests instead of contrast tests [17, 18, 19]. Response‐adaptive extensions of MCP‐Mod with one or multiple interim analyses may lead to power gains to detect a dose–response signal or in higher precision to estimate the dose–response curve or a target dose of interest [20, 21, 22, 23]. More recently Buatois et al. [24], proposed a modification of the MCP‐Mod approach for pharmacometric model‐based analysis of longitudinal phase II dose‐finding studies under model uncertainty [25]. investigated generalized contrast tests to combine dependent *p*‐values, such as Fisher's combination method or the inverse normal combination method [26]. investigated extensions to Bayesian methods that allow for the inclusion of historical data in a systematic fashion. Moreover, MCP‐Mod received a positive qualification opinion by [27] and a fit‐for‐purpose determination by FDA [28].

To the best of our knowledge, however, all these investigations focused mostly on the original scope of MCP‐Mod, that is, Phase II dose finding studies to support dose selection for Phase III. Koenig [29] was the first to extend the MCP‐Mod approach to obtain confirmatory *p*‐values for the global trend assessment (i.e., whether there is any statistical evidence for a dose‐related drug effect) as well as for the pairwise comparisons of the individual doses against placebo in pivotal Phase III trials. In this paper, we formalize these ideas, provide missing proofs for the proposed approach and provide various extensions. To this end, we apply the closed test principle [30] to MCP‐Mod by considering the elementary null hypotheses of comparing the individual doses with placebo. That is, we construct all possible intersection hypotheses on the basis of these elementary hypotheses and define suitable level α tests derived from MCP‐Mod. More specifically, we test each intersection hypothesis using the same set of pre‐specified dose–response models and perform as many contrast tests as models are included in the original candidate set. In doing so, we determine the optimal contrast anew for each intersection hypothesis based on the available doses and subsequently calculate the critical value to control the (local) Type I error rate at level α. Finally, we declare an individual dose to be better than placebo if all intersection hypotheses containing that particular pairwise comparison are rejected by their level α tests, using MCP‐Mod. This approach then controls the FWER at level α in the strong sense according to the closed test principle [30] as long as the contrast coefficients are optimized under suitable constraints. We present the results of a comprehensive simulation study to evaluate the operating characteristics of the proposed method, complementing extending previous simulations results reported by Bretz et al. [31] and Yamaguchi et al. [32]. In addition, we provide an insightful discussion of a neuropathic pain case study and extend the proposed methodology to multiple endpoint scenarios.

The intended use of the proposed approach is in Phase 3 clinical trials with more than two doses, where the main objective is to demonstrate efficacy for an individual dose. Accordingly, this paper is organized as follows. In Section 2, we describe a case study in neuropathic pain from our consultancy experience, which motivates the research in this paper. In Section 3, we describe the core methodology of using modeling approaches to increase the power of declaring effective doses statistically significant. In Section 4, we show that the contrast coefficients have to be optimized under suitable constraints to guarantee strong type 1 error rate control at a pre‐specified significance level α. In Section 5, we present the results of a comprehensive simulation study to evaluate the operating characteristics of the proposed method. In Section 6, we revisit the neuropathic pain case study. In Section 7, we extend the proposed methodology to multiple endpoint scenarios. In Section 8, we discuss further the objectives and the resulting implications for the proposed approach versus dose–response estimation/decision‐making.

## 2 A Case Study in Neuropathic Pain

To motivate this paper, we refer to a Phase III clinical trial of a new drug in patients with neuropathic pain. Four hundred patients are randomized to receive either one of three active dose levels (0.1, 0.4, and 1) or placebo, assuming a balanced allocation. We re‐scaled the original dose levels for confidentiality reasons. A residual standard deviation of 1 is assumed. The primary endpoint of the study is change from baseline of a 24‐h average pain score, using a numeric rating scale, after 12 weeks of treatment. The objective of this study is to establish a dose–response signal and to demonstrate that at least one of the three active doses is statistically significant against placebo. The standard MCP‐Mod approach [9] only allows to test for a dose–response signal but does not make statements about the individual dose‐control comparisons. Since this trial has the dual objective of establishing dose–response and significant treatment effects at the individual doses, while controlling the FWER at a pre‐specified significance level, the standard MCP‐Mod approach needs to be extended accordingly. In the following sections, we describe the closed MCP‐Mod approach for a single, normally distributed endpoint. Extensions to general parametric models following Pinheiro et al. [14] are possible.

**FIGURE 1:**
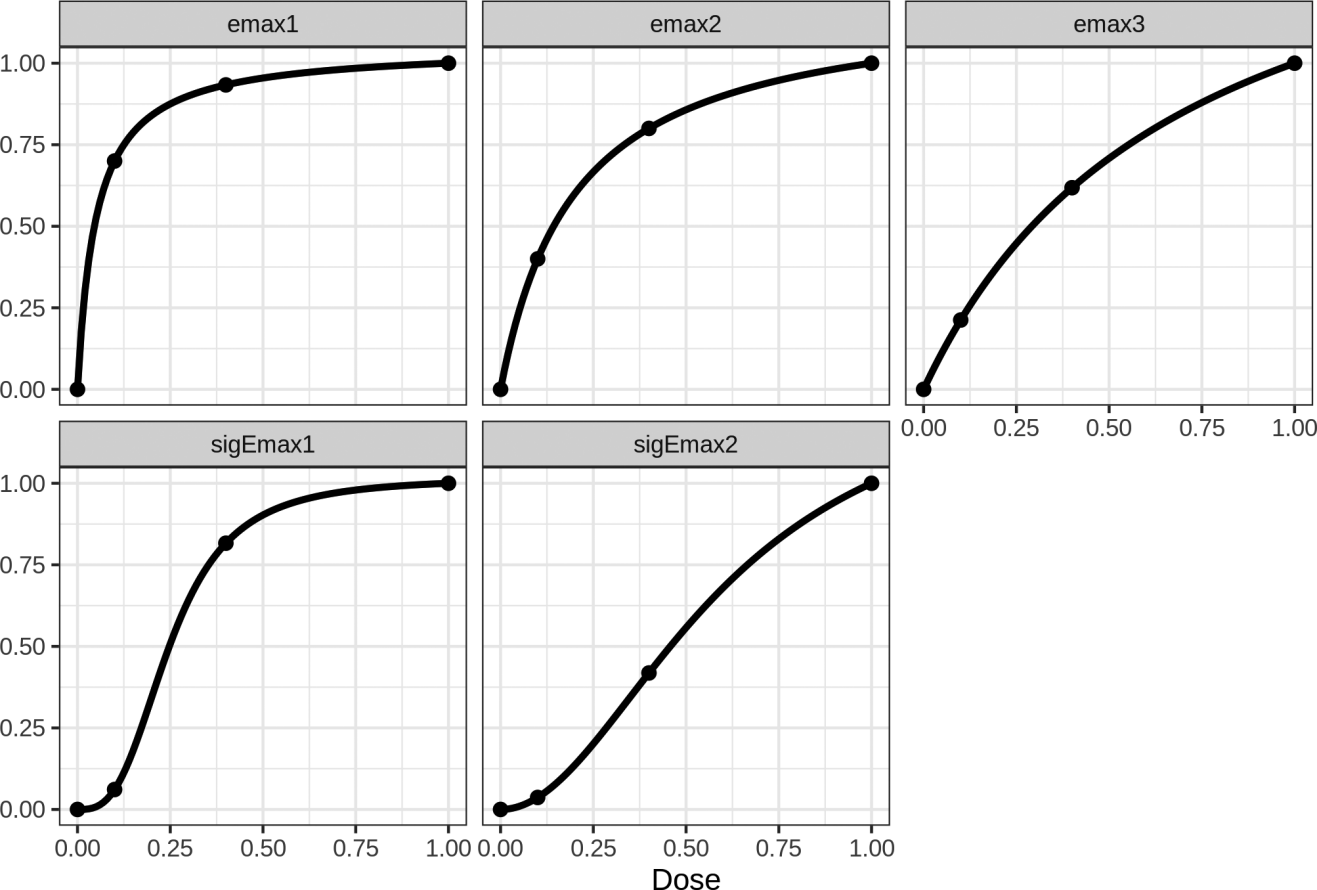
Model shapes for the selected candidate model set.

Five candidate shapes have been identified at the design stage of this neuropathic pain trial. More specifically, we assume the five dose–response shapes summarized in Table 1, namely three shapes derived from an Emax and two from a sigmoid Emax model; see Section 3.1 for the notation used in Table 1. Figure 1 displays the five candidate shapes, scaled so that the placebo effect is 0 and the effect size at the highest dose 1. The major assumption underlying the specified shapes is that of a monotonic dose–response. In addition, we exclude sub‐linear shapes, where both the low and middle dose have no effect, from the candidate model set. We will re‐visit this example for the purpose of motivating the simulation study in Section 5 and illustrating the closed MCP‐Mod approach by means of a numerical example in Section 6.

**TABLE 1:** Model shapes for the selected candidate model set.

Model	Standardized model specifications
Emax1	$d/\left(0.05+d\right)$
Emax2	$d/\left(0.2+d\right)$
Emax3	$d/\left(0.7+d\right)$
sigEmax1	$d^{3}/\left(0.25^{3}+d^{3}\right)$
sigEmax2	$d^{2}/\left(0.6^{2}+d^{2}\right)$

## 3 Methodology

In this section, we describe the extension of MCP‐Mod to obtain confirmatory *p*‐values for detecting a dose–response signal as well as for the pairwise comparisons of individual doses against placebo. In Section 3.1, we introduce the relevant notation. We briefly review the original MCP‐Mod approach in Section 3.2 and the closed test principle in Section 3.3. In Section 3.4, we then describe the core methodology of the closed MCP‐Mod approach.

### 3.1 Notation

Assume that a response Y is observed for a given set of parallel groups of patients corresponding to doses $d_{1},d_{2},\ldots ,d_{k}$ plus placebo $d_{0}$, for a total of k+1 arms. The model is given by

(1) $$Y_{\mathrm{ij}}=f\left(d_{i},\theta \right)+\epsilon_{\mathrm{ij}},\;\epsilon_{\mathrm{ij}}\overset{\mathrm{ind}}{∼}N\left(0,\sigma^{2}\right),\;i=0,\ldots ,k,j=1,\ldots ,n_{i}$$

where the dose–response function $f\left(.\right)$ is parameterized by a parameter vector θ and $\epsilon_{\mathrm{ij}}$ denotes the error term for patient j within dose group i. Most dose–response models used in practice can be expressed as $f\left(d,\theta \right)=\theta_{0}+\theta_{1}f^{0}\left(d,\theta^{0}\right)$. We refer to Pinheiro et al. [11] for an overview of several linear and nonlinear regression dose–response models commonly used in practice. Finally, we let $N=\sum_{i=0}^{k}n_{i}$ and $\mu_{i}=f\left(d_{i},\theta \right),i=0,\ldots ,k$, for the unknown dose–response model $f\left(.\right)$ parameterized through θ.

In confirmatory clinical trials, we are interested in testing the k elementary null hypotheses $H_{i}:\mu_{0}\geq \mu_{i}$ of whether the placebo effect $\mu_{0}$ is larger than or equal to the effect $\mu_{i}$ at dose i=1,…,k. The Dunnett test [5] is a popular approach to compare each dose with a common control. One can conceptually think of Dunnett's method as a set of two‐sample t tests, adjusted for multiplicity but without making any assumptions about the dose–response shape. Alternatively, one can use trend tests by combining the information from neighboring doses, such as the MCP‐Mod trend test described in the next section.

### 3.2 MCP‐Mod

The core idea of MCP‐Mod is to use a set ℳ of M candidate dose–response models $f_{m}\left(d,\theta_{m}\right)$, m=1,…,M, to cover the possible shapes anticipated for the dose–response relationship. For each model $f_{m}\left(.\right)$ we test the associated null hypothesis $H_{0m}:c_{m}^{\prime }\;\mu =0$ using a single contrast test

(2) $$T_{m}=\frac{\sum_{i=0}^{k}c_{\mathrm{mi}}{\bar{Y}}_{i}}{S\sqrt{\sum_{i=0}^{k}c_{\mathrm{mi}}^{2}/n_{i}}},\;m=1,\ldots ,M$$

where

(3) $$S^{2}=\sum_{i=0}^{k}\sum_{j=1}^{n_{i}}{\left(Y_{\mathrm{ij}}-{\bar{Y}}_{i}\right)}^{2}/\left(N-k-1\right)$$

and $c_{m}={\left(c_{m0},\ldots ,c_{\mathrm{mk}}\right)}^{\prime }$ denotes the optimal contrast vector for detecting the *m*th model shape such that $\sum_{i=0}^{k}c_{\mathrm{mi}}=0$. Let $\mu_{m}^{0}={\left(\mu_{m0}^{0},\ldots ,\mu_{\mathrm{mk}}^{0}\right)}^{\prime }={\left(f_{m}^{0}\left(d_{0}\theta_{m}^{0}\right),\ldots ,f_{m}^{0}\left(d_{k}\theta_{m}^{0}\right)\right)}^{\prime }$, where $\;\theta_{m}^{0}$ are the best guesses of the parameters of the standardized models determining the model shapes, to be specified at the trial design stage. Under model (1), the *i*th entry of the optimal contrast $c_{m}$ for detecting the *m*th shape is proportional to

(4) $$n_{i}\left(\mu_{\mathrm{mi}}^{0}-{\bar{\mu }}_{m}\right),\;i=0,\ldots ,k,$$

where ${\bar{\mu }}_{m}=N^{-1}\sum_{i=0}^{k}\mu_{\mathrm{mi}}^{0}n_{i}$ [9, 33]. The optimal contrast is unique only up to a multiplicative constant. To make it unique, we impose the restriction $\sum_{i=0}^{k}c_{\mathrm{mi}}^{2}=1$.

The final test statistic $T_{\max}=\max_{m}\;T_{m}$ is the maximum of all single contrast tests, which follows a multivariate t distribution (for details see Distribution of original MCP‐Mod test statistic). Numerical integration can be used to compute multiplicity adjusted *p*‐values $q_{m}$ and critical values (Genz and Bretz 2002, 2009) [34, 35]. A dose–response signal can hence be established if $T_{\max}\geq c_{1-\alpha }$, where $c_{1-\alpha }$ denotes the multiplicity adjusted critical value from the multivariate t distribution. Equivalently, this decision rule can be stated in terms of multiplicity adjusted *p*‐values $q_{m}$ so that a dose–response signal can be established if $\min_{m}\;q_{m}<\alpha$.

If no candidate model is statistically significant, the procedure stops, indicating that a dose–response relationship cannot be established from the observed data. Otherwise, all dose–response shapes with contrast test statistics larger than $c_{1-\alpha }$ are declared statistically significant at level α and standard model selection criteria or model averaging techniques can be employed based on the individual model fits to obtain a dose–response and target dose estimate [36].

Software implementations of MCP‐Mod are available in both R (DoseFinding package [37]) and SAS [38]. We refer to Verrier et al. [39] and Xun and Bretz [12] for practical considerations on the use of MCP‐Mod.

### 3.3 Closed Testing

We now briefly describe closed test procedures [30] as they form the basis of the closed MCP‐Mod extension introduced in Section 3.4. Assume that we test k elementary null hypotheses $H_{1},\ldots ,H_{k}$. For example, in the setting of Section 3.1, we are interested in testing the k hypotheses $H_{i}:\mu_{0}\geq \mu_{i},i=1,\ldots ,k$. The closed test principle is a general approach to construct multiple test procedures that control the FWER in the strong sense at level α. That is, the probability of rejecting at least one true null hypothesis is bounded by α under any configuration of true and false null hypotheses. Following the closed test principle, we test all possible intersection hypotheses based on the initial set of elementary hypotheses. We then reject an elementary null hypothesis if all intersection hypotheses formed by intersection with that particular elementary hypothesis are rejected by their *α*‐level tests. In other words, a closed test procedure proceeds as follows:

1. Define the family of elementary null hypotheses $H_{1},\ldots ,H_{k}$;
2. construct all intersection hypotheses $H_{I}=\cap_{i\in I}H_{i},I\subseteq \left\{1,\ldots ,k\right\}$;
3. define an *α*‐level test for every intersection hypothesis $H_{I}$;
4. reject an elementary hypothesis $H_{i}$, if all hypotheses $H_{I}$ with i∈I are rejected by their *α*‐level tests.

Any closed test procedure controls the FWER in the strong sense, regardless of the choice for the intersection hypothesis tests. For example, employing the Dunnett test for each intersection hypothesis $H_{I}$ results in the step‐down Dunnett test [6].

### 3.4 Closed MCP‐Mod for Pairwise Comparisons of Several Doses With a Control

We now apply the closed test principle from Section 3.3 to the k elementary null hypotheses $H_{i}:\mu_{0}\geq \mu_{i},i=1,\ldots ,k$, using the MCP‐Mod contrast tests from Section 3.2 for each intersection hypothesis. To this end, we use the same set of pre‐specified candidate models, but recalculate the optimal contrasts based on the set of doses that remain in a given intersection hypothesis. The resulting closed MCP‐Mod approach thus extends the original MCP‐Mod approach in that it provides multiplicity adjusted *p*‐values for the pairwise comparisons of dose i against placebo, i=1,…,k, while still using contrasts tests for the intersection hypotheses. In particular, the original MCP‐Mod test $T_{\max}$ from Section 3.2 is applied to the global intersection hypothesis $H_{1}\cap \ldots \cap H_{k}$. Thus, the closed MCP‐Mod can be interpreted as starting with the original MCP‐Mod test as a gatekeeper, and only if a significant dose–response signal can be established it continues with the remaining closed test to compute the multiplicity adjusted *p*‐values for the pairwise comparisons.

Tailoring the closed test principle from Section 3.3 to our needs, we can describe the closed MCP‐Mod approach operationally as follows:

1. Define the family of elementary null hypotheses $H_{i}:\mu_{0}\geq \mu_{i},i=1,\ldots ,k$;
2. construct all intersection hypotheses $H_{I}=\cap_{i\in I}H_{i},I\subseteq \left\{1,\ldots ,k\right\}$;
3. define a set ℳ of pre‐specified candidate dose–response models as basis for testing $H_{I}$;
4. test $H_{I}$ using MCP‐Mod contrast tests by determining anew the optimal contrasts for each candidate model based on the doses $d_{j}$ with j∈J=0∪I;
5. reject $H_{i}$ if all hypotheses $H_{I}$ with i∈I are rejected by their respective *α*‐level tests.

When testing $H_{I}$ in Step 4, we define the single contrast test for the *m*th model similar to (2) but using only the doses $d_{j}$, j∈J=0∪I. The modified test statistic for the *m*th model and intersection index set I is given by

(5) $$T_{m,I}=\frac{\sum_{j\in J}c_{\mathrm{mj},I}{\bar{Y}}_{j}}{S\sqrt{\sum_{j\in J}c_{\mathrm{mj},I}^{2}/n_{j}}},\;m=1,\ldots ,M$$

where $c_{m,I}={\left(c_{m0,I},c_{m1,I},\ldots ,c_{\mathrm{mk},I}\right)}^{\prime }$ is the optimal contrast vector for detecting model shape m with $\sum_{j\in J}c_{\mathrm{mj},I}=0$ and $\sum_{j\in J}c_{\mathrm{mj},I}^{2}=1$. The coefficients $c_{\mathrm{mj},I},j\in J=0\cup I,$ are the optimal contrasts for testing model shape m using only the doses i∈I and the control group i=0. In Section 4, we show that the contrast coefficients have to be optimized under suitable constraints to guarantee Type 1 error rate control at a pre‐specified significance level α. For any j∉J we set $c_{\mathrm{mj},I}=0$. Note that we continue using the pooled variance estimate (3).

The final test statistic $T_{\max,I}=\max_{m}\;T_{m,I}$ for $H_{I}$ is the maximum of all single contrast tests $T_{m,I}$. Its distribution can be derived from the joint distribution of $\;T={\left(T_{1,I},\ldots ,T_{M,I}\right)}^{\prime }$, which follows again a central multivariate t distribution, but using a modified correlation matrix $R_{I}$ for each $H_{I}$ (for details see Distribution of closed MCP‐Mod test statistic). As for the original MCP‐Mod approach defined in Section 3.2, we compute multiplicity adjusted *p*‐values $q_{m,I}$ and critical values $c_{1-\alpha ,I}$ using the modified correlation structure $R_{I}$ of the contrast tests defined for $H_{I}$. An intersection hypothesis $H_{I}$ can hence be rejected if $T_{\max,I}\geq c_{1-\alpha ,I}$ or, equivalently, $\min_{m}\;q_{m,I}<\alpha$.

As mentioned before, the final test statistic for the global null hypothesis $\cap_{i=1}^{k}H_{i}$ reduces to the one from the original MCP‐Mod approach described in Section 3.2 as it uses the data from all doses i=1,…,k and control i=0. For the elementary null hypotheses $H_{i}$, i=1,…,k, the contrast coefficients collapse to the same coefficients $c_{m0,I}=-c_{\mathrm{mi},I}$ for $I=\left\{i\right\}$ and across all candidate models m∈ℳ, with the understanding that $c_{\mathrm{mj},I}=0,j\notin 0\cup I$.

## 4 Derivation of Optimal Contrast Coefficients

In this section, we discuss the choice of contrast coefficients. In Section 4.1, we discuss the impact on the Type I error rate when testing an intersection hypothesis $H_{I}=\cap_{i\in I}H_{i},I\subseteq \left\{1,\ldots ,k\right\}$ for the one‐sided elementary null hypotheses $H_{i}:\mu_{0}\leq \mu_{i}$ $\left(i=1,\ldots ,k\right)$. In Section 4.2, we then derive optimal contrast coefficients that are constrained such that the Type I error rate is controlled under any configuration of $\mu_{0},\mu_{1},\ldots ,\mu_{k}$.

### 4.1 Type I Error Rate Control

MCP‐Mod was initially developed for exploratory dose finding trials to assess whether the dose–response curve is flat. More specifically, it assesses whether $\mu_{0}=\mu_{1}=\ldots =\mu_{k}$; if rejected, it concludes in favour of a dose–response signal. Thus, the original MCP‐Mod approach controls the Type I error rate when $\mu_{0}=\mu_{1}=\ldots =\mu_{k}$ and the proposed closed MCP‐Mod procedure weakly controls the FWER at level α. However, if in reality $\mu_{i}<\mu_{0}$ holds for some i>0, a misspecification of the dose–response profile (with some negative weights $c_{\mathrm{mi},H_{I}}$ for i>0) might lead to situations where an intersection $H_{I}$ is rejected with probability larger than α. Consider, for example, a linear dose–response shape with four equidistant doses (including placebo) and contrasts coefficients −0.67,−0.22,0.22,0.67. Assume $\mu_{0}=\mu_{2}=\mu_{3}>\mu_{1}$ (i.e., the lowest dose does harm). If $\mu_{1}$ goes to −∞, the probability of the linear contrast test to reject the null hypothesis of no treatment effect will converge to 1 (even though the null hypothesis is true). This is an intrinsic feature of the underlying contrast tests (and thus the original MCP‐Mod procedure) and needs to be considered when using the closed MCP‐Mod approach.

This toy example illustrates that the contrast tests from the original MCP‐Mod procedure may not control the Type I error rate for a given intersection hypothesis $H_{I}$ under all possible configurations of the $\mu_{i}$. However, when applying the closed test procedure, the FWER might still be controlled in a strong sense. For example, in the toy example the probability of rejecting the elementary null hypothesis $H_{1}:\mu_{1}\leq \mu_{0}$ will approach 0 for $\mu_{1}\to -\infty$. In Test of two doses against a comparator with a global test using optimal weights, we prove more formally that the FWER is strongly controlled when using the closed MCP‐Mod approach for k=2.

There are several ways to guarantee that the closed MCP‐Mod procedure based on the one‐sided elementary hypotheses $H_{i}:\mu_{0}\leq \mu_{i}$ controls the FWER at level α in the strong sense. For example, if one is willing to assume $\mu_{i}\geq \mu_{0}$ for all i>0, then strong FWER control is guaranteed. Strong control is also enforced if we allow only combinations of candidate models and doses which result in non‐negative weights $c_{\mathrm{mi},H_{I}}\geq 0$ for i>0. Finally, if one is not willing to make these restrictions and assumptions, one can impose the constraints $c_{\mathrm{mi},H_{I}}\geq 0$ for i>0 when determining optimal contrasts for any candidate model m for testing $H_{I}$. This contrained MCP‐Mod contrast test is introduced in the next section.

### 4.2 Constrained MCP‐Mod: Proposal for Alternative Test in MCP‐Mod Replacing the “Optimal Contrast” Test

In this section, we describe how to derive constrained contrasts for the global test. The same principles apply to calculating them for any test of an intersection hypothesis which occurs in the closed test procedure. The MCP test from Section 3.2 is based on optimal contrasts $c_{m}$ which maximize the (assumed) non‐centrality parameter $\frac{{\left(c_{m}^{\prime }\mu_{m}^{0}\right)}^{2}}{c_{m}^{\prime }A_{m}c_{m}}$ of a multivariate *t*‐distribution. The constraint $c_{m}^{\prime }1_{k+1}=0$ is imposed. The contrasts are subsequently used to test $H_{0m}:c_{m}^{\prime }\mu =0$ versus the alternative $c_{m}^{\prime }\mu >0$. Here, $A_{m}$ denotes the estimated covariance matrix of the estimate of $\mu =\left(\mu_{0},\ldots ,\mu_{k}\right)$. (In this simple case, if $n_{0}=n_{1}=\ldots =n_{k}$, $A_{m}$ is just the identity matrix. In more complicated cases with covariates, it is proportional to the relevant submatrix of ${\left(X_{m}^{\prime }X_{m}\right)}^{-1}$ where $X_{m}$ is the design matrix containing the covariates.) As illustrated in the previous section, a misspecification of the assumed profile $\mu_{m}^{0}$ might lead to situations where this test rejects with a probability larger than the nominal level α, if in reality all $\mu_{i}\leq \mu_{0}$ holds for all i>0.

Our proposal is to replace the “optimal contrasts” by a different construction in two‐stages:

1. Calculate contrasts with placebo: $K\mu_{m}^{0}$ where $K=\left(-1_{k}\vdots I_{k}\right)$.
2. Find “optimal weights” $w_{m}=\left(w_{m1},\ldots ,w_{\mathrm{mk}}\right)$ such that

(6) $$\frac{w_{m}^{\prime }K\mu_{m}^{0}}{\sqrt{w_{m}^{\prime }{\mathrm{KA}}_{m}K^{\prime }w_{m}}}$$

is maximized under the additional constraint $w_{\mathrm{mi}}\geq 0$ for all i=1,…,k.

In Calculation of optimal weights with constraints we show that without the constraint, the solution of (6) is identical to the originally suggested optimal contrasts from Bretz et al. [9], which can be used for deriving the contrasts. The calculation of these contrasts is implemented in R‐packages DoseFinding [37] using the option “type = constrained” in the optContr function. Compared to the unconstrained contrasts, the constrained contrasts typically set the coefficients of doses with “weak” effects to 0. The advantage of this approach is that it yields a genuinely one‐sided test that does not “borrow strength” from coupling a negative $c_{i}$ with a negative response ${\bar{y}}_{i}$ on a dose i.

For the constrained closed MCP‐Mod procedure, for all intersection hypotheses $H_{I},I\subseteq \left\{1,\ldots ,k\right\}$ and dose–response models m, minℳ, constrained coefficient are calculated instead of the originally suggested “unconstrained” contrasts by Bretz et al. [9].

## 5 Simulation Study

In this section, we report the results of a simulation study to illustrate the methods described previously. In Section 5.1, we describe the design of the simulation study, including its assumptions and scenarios as well as the performance metrics used to evaluate different operating characteristics of the various methods. In Section 5.2, we summarize the results.

### 5.1 Design of Simulation Study

As described in Section 2, we consider three active doses 0.1,0.4,1 and placebo. We assumed that in total 400 patients are randomized equally to the four treatment groups. For MCP‐Mod we use the candidate models described in Table 1.

**FIGURE 2:**
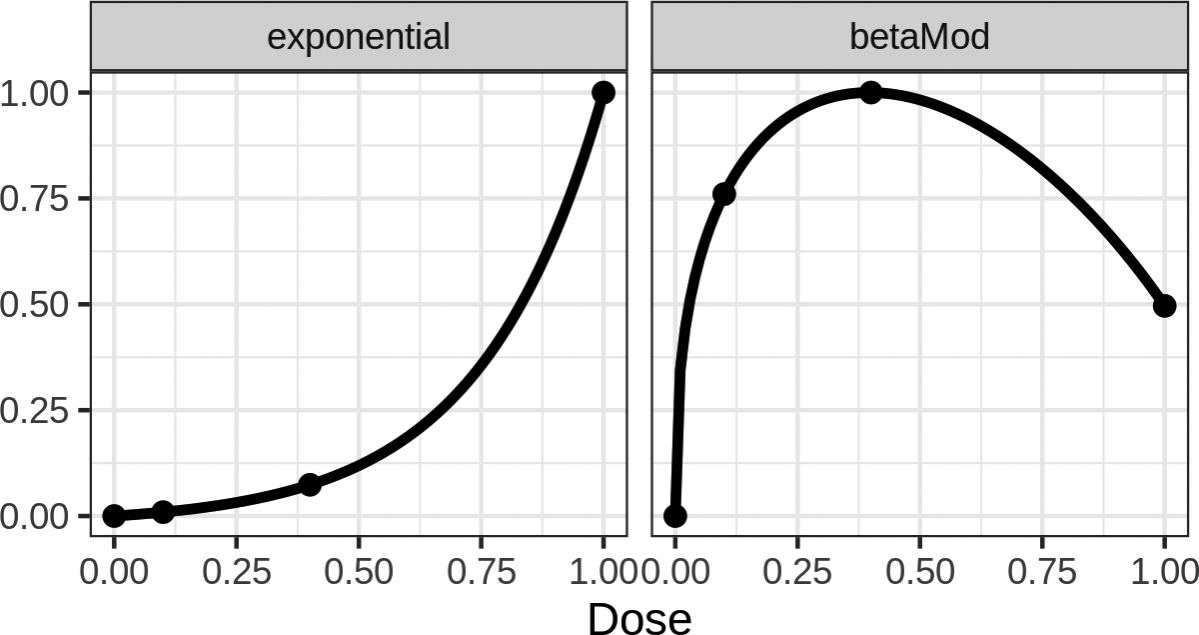
Model shapes not in a set of candidate models.

For data‐generation we consider seven dose–response shapes and three different effect sizes. The first five models are the candidate models used for MCP‐Mod (from Table 1, see also Figure 1). In addition two further models are utilized: The beta and exponential models (betaMod, exponential) with model equations proportional to ${\left(d/1.2\right)}^{0.37}{\left(1-d/1.2\right)}^{0.75}$ and $\exp\left(d/0.25\right)$. In Figure 2 those are displayed (rescaled) so that the highest effect is 1. These models are included in the simulation to investigate the performance of the MCP‐Mod procedures under mis‐specification of the model set.

In addition to the seven dose–response shapes, three different effect sizes are assumed, namely 0.3, 0.4, and 0.5. In each case the dose–response models parameters were calculated to achieve an effect of 0 at placebo and the desired maximum effect at the dose that provides maximum efficacy, which is equal to dose 1 for all models apart from the beta model, where the maximum effect is achieved at dose 0.4. A residual standard deviation of 1 is used, and each simulation scenario has been repeated 10 000 times.

### 5.2 Simulation Results

We compared the results of closed MCP‐Mod approach with the step‐down Dunnett test, which is a short cut for the closed Dunnett test, where Dunnett tests are used for testing every intersection hypothesis constructed according to the closed testing procedure. We also used a fixed sequence test for testing elementary hypotheses based on *t*‐tests as a competitor to our approach. Here testing of the hypotheses follows a fixed order from highest to lowest dose at an unadjusted level. If a comparison cannot be rejected the procedure stops and the remaining elementary hypotheses cannot be rejected. In addition the Hochberg procedure is included in the comparison. Closed MCP‐Mod is evaluated in two variants, once with unconstrained and once with constrained contrasts.

We compare the approaches with respect to the power to reject at least one hypothesis (RAO), the power to reject the individual hypotheses, as well as the average number of rejections divided by three (AVE/3).

**FIGURE 3:**
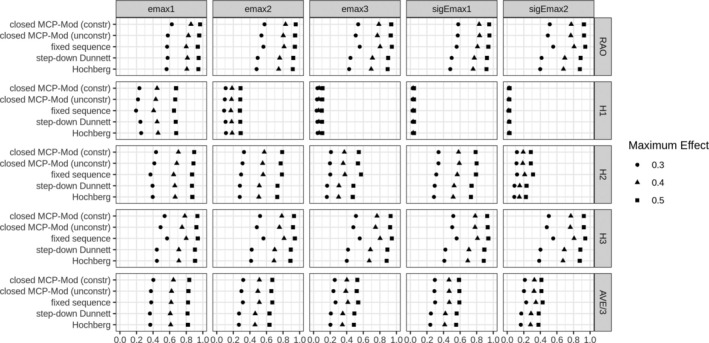
Results of the simulation study with the sampling model included in a set of candidate models (Figure 1). At the horizontal axis, we have power, on the vertical axis approaches used. RAO is the power to reject at least one hypothesis. AVE/3 the average number of rejections divided by 3.

If the true sampling model is included in the set of candidate models (Figure 3), the closed MCP‐Mod approaches outperform the other approaches for most scenarios and metrics. Only the fixed sequence test performs similarly or slightly better in some scenarios. The simulation results also show that the closed MCP‐Mod approach with the constrained weights outperforms the unconstrained closed MCP‐mod. It can be explained due to the fact that original MCP‐Mod was designed for testing PoC and no additional constrains are required for contrasts. As one reviewer pointed out, the same parameter specifications of the sigmoid Emax model [40, 41] were used to generate both the contrasts and the simulated data, excluding the data simulated from the exponential and beta models. We note that even if the conjectured parameters are not precisely the same, the power is maintained due to the high correlation between the contrast test statistics, as discussed in, for example [11], when establishing proof of concept with the original MCP‐Mod approach.

**FIGURE 4:**
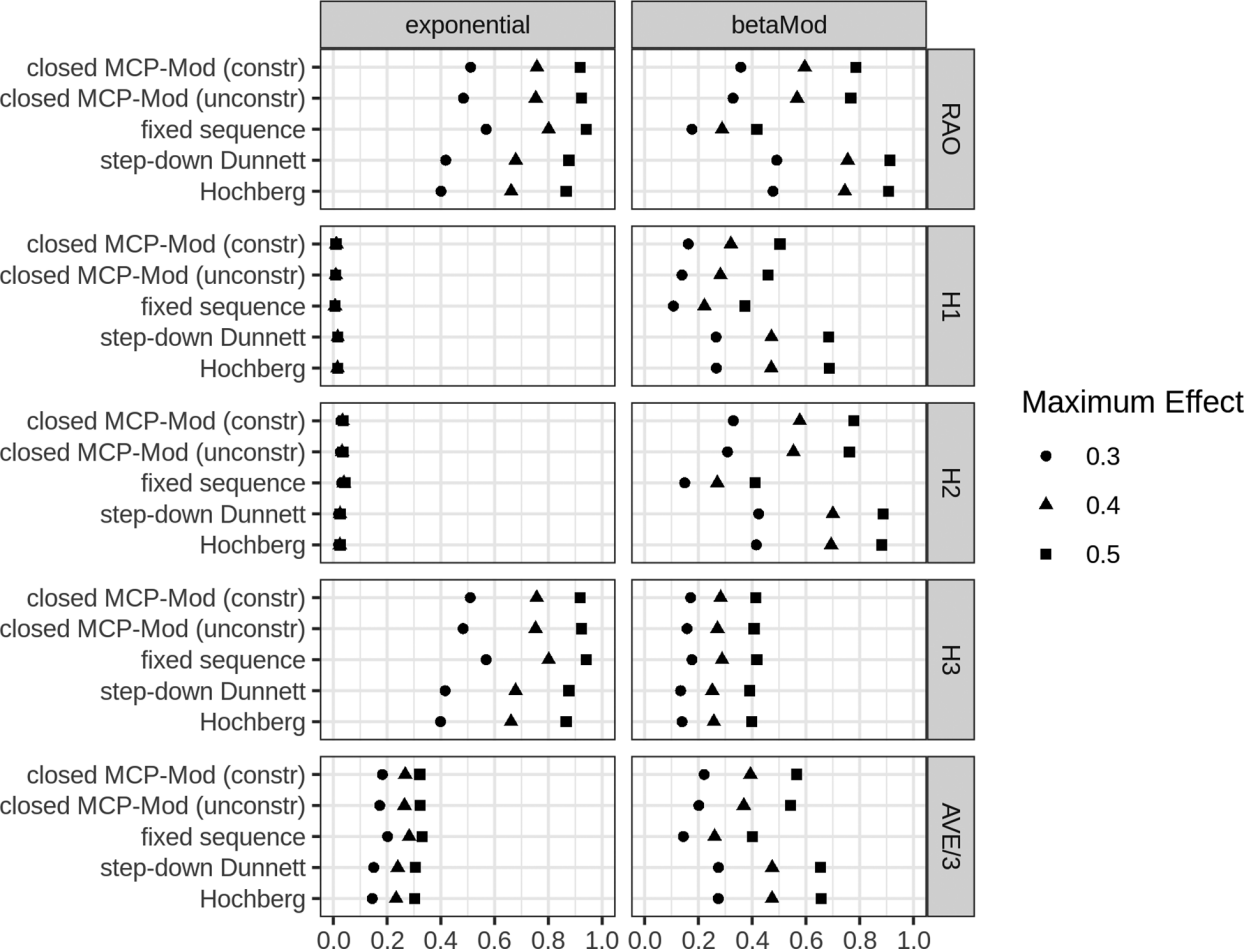
Results of the simulation study with the sampling model not included in a set of candidate models (Figure 2). At the horizontal axis, we have power, on the vertical axis approaches used—Hierarchical, closed MCP‐Mod, constrained and unconstrained options, and closed Dunnett. Different values of the effect size are used.

In cases, where the candidate models are misspecified (Figure 4), we see that the closed MCP‐Mod approach is performing worse than competing approaches. While it still performs competitive for the exponential scenario, there is a larger loss of power for the beta model. The performance of the closed MCP‐Mod approach is better compared to the fixed sequence test in the beta model scenario. Even though both approaches make an assumption of monotonicity, the closed MCP‐Mod procedures appear to be less sensitive to this assumption.

## 6 Neuropathic Pain Case Study Revisited

We now revisit the neuropathic pain case study from Section 2. For that purpose we utilize a simulated dataset using n=100 patients per dose. We are interested in testing three active dose levels $d_{1}=0.1$, $d_{2}=0.4$ and $d_{3}=1$ against placebo $d_{0}=0$ testing elementary null hypotheses $H_{1},H_{2}$ and $H_{3}$, respectively. We show the optimal unconstrained and constrained contrasts for the different elementary and intersection hypotheses in Tables 2 and 3.

**TABLE 2:** Optimal unconstrained contrasts for the different models and intersection hypotheses.

	Emax1	Emax2	Emax3	sigEmax1	sigEmax2
H1_1,2,3_	(−0.831, 0.053, 0.347, 0.431)	(−0.716, −0.195, 0.325, 0.586)	(−0.596, −0.319, 0.209, 0.706)	(−0.528, −0.46, 0.391, 0.597)	(−0.452, −0.407, 0.068, 0.791)
H_1,2_	(−0.793, 0.226, 0.566, 0)	(−0.707, 0, 0.707, 0)	(−0.623, −0.145, 0.768, 0)	(−0.455, −0.36, 0.815, 0)	(−0.463, −0.351, 0.814, 0)
H_1,3_	(−0.781, 0.184, 0, 0.597)	(−0.656, −0.094, 0, 0.749)	(−0.542, −0.257, 0, 0.8)	(−0.446, −0.369, 0, 0.815)	(−0.431, −0.385, 0, 0.816)
H_2,3_	(−0.815, 0, 0.365, 0.45)	(−0.802, 0, 0.267, 0.535)	(−0.756, 0, 0.11, 0.645)	(−0.804, 0, 0.28, 0.524)	(−0.666, 0, −0.077, 0.742)
H_1_	(−0.707, 0.707, 0, 0)	(−0.707, 0.707, 0, 0)	(−0.707, 0.707, 0, 0)	(−0.707, 0.707, 0, 0)	(−0.707, 0.707, 0, 0)
H_2_	(−0.707, 0, 0.707, 0)	(−0.707, 0, 0.707, 0)	(−0.707, 0, 0.707, 0)	(−0.707, 0, 0.707, 0)	(−0.707, 0, 0.707, 0)
H_3_	(−0.707, 0, 0, 0.707)	(−0.707, 0, 0, 0.707)	(−0.707, 0, 0, 0.707)	(−0.707, 0, 0, 0.707)	(−0.707, 0, 0, 0.707)

*Note*: Contrast have been scaled to have norm 1.

**TABLE 3:** Optimal constrained contrasts for the different models and intersection hypotheses.

	Emax1	Emax2	Emax3	sigEmax1	sigEmax2
H_1,2,3_	(−0.831, 0.053, 0.347, 0.431)	(−0.802, 0, 0.267, 0.535)	(−0.756, 0, 0.11, 0.645)	(−0.804, 0, 0.28, 0.524)	(−0.707, 0, 0, 0.707)
H_1,2_	(−0.793, 0.226, 0.566, 0)	(−0.707, 0, 0.707, 0)	(−0.707, 0, 0.707, 0)	(−0.707, 0, 0.707, 0)	(−0.707, 0, 0.707, 0)
H_13_	(−0.781, 0.184, 0, 0.597)	(−0.707, 0, 0, 0.707)	(−0.707, 0, 0, 0.707)	(−0.707, 0, 0, 0.707)	(−0.707, 0, 0, 0.707)
H_2,3_	(−0.815, 0, 0.365, 0.45)	(−0.802, 0, 0.267, 0.535)	(−0.756, 0, 0.11, 0.645)	(−0.804, 0, 0.28, 0.524)	(−0.707, 0, 0, 0.707)
H_1_	(−0.707, 0.707, 0, 0)	(−0.707, 0.707, 0, 0)	(−0.707, 0.707, 0, 0)	(−0.707, 0.707, 0, 0)	(−0.707, 0.707, 0, 0)
H_2_	(−0.707, 0, 0.707, 0)	(−0.707, 0, 0.707, 0)	(−0.707, 0, 0.707, 0)	(−0.707, 0, 0.707, 0)	(−0.707, 0, 0.707, 0)
H_3_	(−0.707, 0, 0, 0.707)	(−0.707, 0, 0, 0.707)	(−0.707, 0, 0, 0.707)	(−0.707, 0, 0, 0.707)	(−0.707, 0, 0, 0.707)

*Note*: Contrast have been scaled to have norm 1.

As can be seen, for testing the global hypothesis $H_{\left\{1,2,3\right\}}$, all models and four doses, including placebo, are used. For testing the hypothesis $H_{\left\{2,3\right\}}$ again all candidate models are used, but now three doses, including placebo, are used. The doses not included in the intersection hypothesis obtain a 0 in the contrast. For testing a single elementary hypothesis, for example, $H_{3}$ using MCP‐Mod approach, the optimal contrast reduces to a pairwise comparison (a simple *t*‐test) for every candidate model. The fewer doses are contained in an intersection hypothesis, the more similar the shapes of the contrast coefficients become between models. We apply the same procedure for every intersection hypothesis. In the end, we reject an elementary hypothesis only if all intersection hypotheses that contain that particular hypothesis are rejected.

Comparing the constrained and unconstrained optimal contrasts in Tables 2 and 3, it becomes clear that constrained contrasts contain only positive coefficients for the active doses (as expected by construction). When the unconstrained optimal contrast does not contain a negative contrast coefficient for the active doses, constrained and unconstrained optimal contrasts are equal. But generally more zeros appear in the constrained contrasts. Some of the intersection hypotheses even reduce to pairwise comparisons for some candidate models.

Tables 4 and 5 contain the *p*‐values for the different models under the different hypotheses (the *p*‐values are adjusted row‐wise). The minimum *p*‐value per row represents the *p*‐value for the corresponding intersection hypothesis. It can be seen that for the global null hypothesis the unconstrained contrasts provide a slightly smaller *p*‐value, while for the intersection hypotheses involving two doses the constrained contrasts appear to perform better. For the elementary hypotheses both type of contrasts obviously provide the same result.

**TABLE 4:** Adjusted *p*‐values for all intersection hypotheses and all candidate models under the unconstrained contrasts.

	Emax1	Emax2	Emax3	sigEmax1	sigEmax2
H_1,2,3_	0.0123	0.0049	0.0045	0.0036	0.0065
H_1,2_	0.0723	0.0416	0.0338	0.0330	0.0328
H_1,3_	0.0240	0.0089	0.0070	0.0072	0.0072
H_2,3_	0.0079	0.0074	0.0083	0.0073	0.0133
H_1_	0.3492	0.3492	0.3492	0.3492	0.3492
H_2_	0.0247	0.0247	0.0247	0.0247	0.0247
H_3_	0.0065	0.0065	0.0065	0.0065	0.0065

*Note*: The minimum *p*‐value per row is the *p*‐value for the corresponding intersection hypothesis.

**TABLE 5:** Adjusted *p*‐values for all intersection hypotheses and all candidate models under the constrained contrasts. The minimum *p*‐value per row is the *p*‐value for the corresponding intersection hypothesis.

	Emax1	Emax2	Emax3	sigEmax1	sigEmax2
H_1,2,3_	0.0092	0.0069	0.0078	0.0068	0.0098
H_1,__2_	0.0553	0.0311	0.0311	0.0311	0.0311
H_1,3_	0.0171	0.0083	0.0082	0.0083	0.0081
H_2,3_	0.0073	0.0068	0.0078	0.0067	0.0100
H_1_	0.3492	0.3492	0.3492	0.3492	0.3492
H_2_	0.0247	0.0247	0.0247	0.0247	0.0247
H_3_	0.0065	0.0065	0.0065	0.0065	0.0065

In Table 6, one can observe the results of closed MCP‐Mod based on unconstrained and constrained contrasts, in addition the Hochberg, step‐down Dunnett adjusted *p*‐values are shown. For reference the unadjusted *p*‐values are also included (which are in this case equal to the *p*‐values that would originate from the fixed sequence test).

**TABLE 6:** *p*‐values for the pairwise comparisons under different ways of adjusting for multiplicity.

Hypothesis	Unadjusted	Bonferroni	Hochberg	step.down.Dunnett	Unconstrained	Constrained
H_1_ (0.1 vs. PBO)	0.3492	1.0000	0.3492	0.3492	0.3492	0.3492
H_2_ (0.4 vs. PBO)	0.0247	0.0742	0.0495	0.0449	0.0330	0.0311
H_3_ (1 vs. PBO)	0.0065	0.0196	0.0196	0.0176	0.0072	0.0081

*Note*: The unadjusted *p*‐values are also included as a reference.

## 7 Extension to a Primary and a Secondary Endpoint

In this section, we briefly discuss how to extend the closed MCP‐Mod procedure from testing a single endpoint to testing a primary and a secondary endpoint. This extension is also motivated by the case study in Section 2 where the pain score is the primary endpoint and a secondary endpoint is patients' quality of life.

To facilitate the discussion, let $H_{1i}:\mu_{1i}\leq \mu_{10}$ be the primary hypothesis and $H_{2i}:\mu_{2i}\leq \mu_{20}$ be the secondary hypothesis of the dose $d_{i},i=1,\ldots ,k$ against placebo $d_{0}$. In theory, two different sets of candidate models could be prespecified respectively for the primary and the secondary endpoint, but we assume a common set for simplicity. Following the closed testing principle in Section 3.3, the FWER is controlled at level α if every intersection hypothesis is tested at level α. If the intersection hypothesis only involves either primary hypotheses or secondary hypotheses, we can apply the constrained MCP‐Mod approach in Section 4 at level α based on all candidate models and the available doses for that intersection.

The key to the extension is how to test the intersection hypothesis which involves both primary and secondary hypotheses. Because the correlation between the primary and the secondary endpoint is usually not known or difficult to quantify at the design stage, we adopt the idea from [42] to test the intersection hypothesis first using a Bonferroni split between the group of primary hypotheses and the group of secondary hypotheses, and then to test each group using separate constrained MCP‐Mod approaches. To determine the Bonferroni split, we will use the graphical approach by Bretz et al. [43]. In addition, because the primary endpoint is usually more important in determining the trial success, we require that the secondary hypothesis is tested only if the corresponding primary hypothesis on the same dose has been rejected. This logical restriction on the two endpoints can be easily incorporated into the graphical approach. We illustrate the idea of the graphical approach using the case study from Section 2.

**FIGURE 5:**
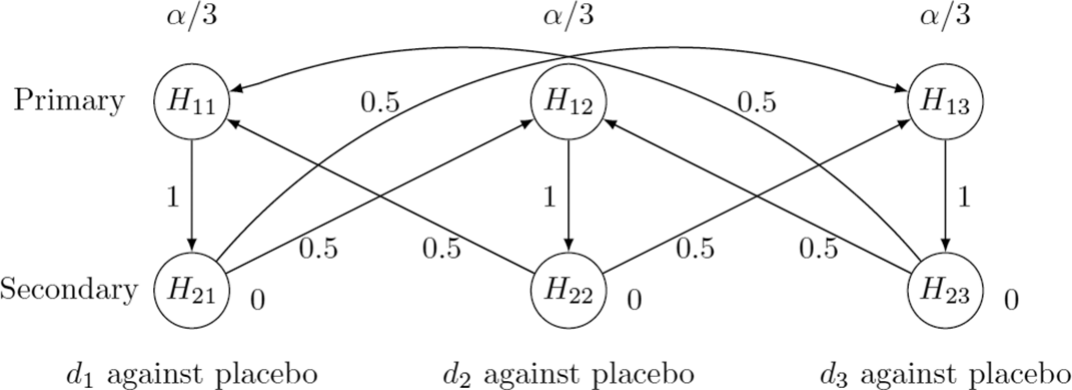
Graphical multiple test procedure for a primary and a secondary endpoint with three dose levels against placebo.

In the graphical approach, hypotheses are denoted by nodes associated with their local significance level. A directed edge from a hypothesis to another hypothesis represents how the local significance level is propagated if the hypothesis on the origin is rejected. The graphical representation of the testing strategy is shown in Figure 5, whereby the same testing strategy has be shown in fig. 8 in Bretz et al. [43]. Because the three dose levels are equally important for the contrast test, we split the total level α equally among primary hypotheses. A directed edge with the transition weight 1 from $H_{1i}$ to $H_{2i},i=1,2,3$ means that when the primary hypothesis is rejected, its local significance level can be propagated to the corresponding secondary hypothesis, which can be tested at the same level. When a secondary hypothesis is rejected, its local significance level will be divided equally and propagated to the primary hypotheses of the other two dose levels. These create a symmetric graph among three dose‐control comparisons and thus allow the MCP‐Mod approach to be implemented using the total significance level of hypotheses involved [44].

It has been shown that the graphical approach defines a closed testing procedure and thus controls the FWER strongly at level *α* [43]. Algorithm 1 in [45] defines the local significance level of elementary hypotheses for each intersection hypothesis. Due to the limited space, we provide some representative cases to illustrate the idea in Table 7. We can see that if all primary and/or any secondary hypothesis are involved (e.g., the overall intersection hypothesis $\cap_{i=1,2,3}H_{1i}\cap_{i=1,2,3}H_{2i}$), no secondary hypothesis has a positive local level. Thus we can test $\cap_{i=1,2,3}H_{1i}$ using the constrained MCP‐Mod approach at the sum of the significance level of the primary hypotheses, which is α. This is also the case when only secondary hypotheses are involved (e.g., $\cap_{i=1,2,3}H_{2i}$). If two primary and all secondary hypotheses are involved (e.g., $\cap_{i=1,2}H_{1i}\cap_{i=1,2,3}H_{2i}$), the local significance level for $H_{11}$, $H_{12}$ and $H_{23}$ is α/3 and 0 for other hypotheses. Thus we can test $H_{11}\cap H_{12}$ using the constrained MCP‐Mod test at level 2α/3 and $H_{23}$ at level α/3 with the Bonferroni split. If two primary and their corresponding secondary hypotheses are involved (e.g., $\cap_{i=1,2}H_{1i}\cap_{i=1,2}H_{2i}$), the local level for $H_{11}$ and $H_{12}$ is α/2 and 0 for any other hypothesis. Thus we can test $H_{11}\cap H_{12}$ at level α. If one primary and all secondary hypotheses are involved (e.g., $H_{11}\cap_{i=1,2,3}H_{2i}$), the local level for $H_{11}$, $H_{22}$ and $H_{23}$ is α/3. Thus we can test $H_{22}\cap H_{23}$ at level 2α/3 and $H_{11}$ at level α/3 with the Bonferroni split. If one primary hypothesis and a secondary hypothesis of another dose are involved (e.g., $H_{11}\cap H_{23}$), the local level for $H_{11}$ and $H_{23}$ is α/2. Thus we can test $H_{11}$ and $H_{23}$ separately at level α/2.

**TABLE 7:** Local significance level of elementary hypotheses for intersection hypotheses based on Figure 5.

		Local significance level					
Hypothesis involved	Intersection	$H_{11}$	$H_{12}$	$H_{13}$	$H_{21}$	$H_{22}$	$H_{23}$
All primary and/or secondary	11, 12, 13, 21, 22, 23	α/3	α/3	α/3	0	0	0
All secondary	21, 22, 23	—	—	—	α/3	α/3	α/3
Two primary and all secondary	11, 12, 21, 22, 23	α/3	α/3	—	0	0	α/3
Two primary and two secondary	11, 12, 21, 22	α/2	α/2	—	0	0	—
A primary and all secondary	11, 21, 22, 23	α/3	—	—	0	α/3	α/3
A primary and two secondary	11, 21, 23	α/2	—	—	0	—	α/2
A primary and a secondary	11, 23	α/2	—	—	—	—	α/2

In general, we have the following steps to test each intersection hypothesis $H_{I}$:

1. Let $I_{1}^{\prime }$ and $I_{2}^{\prime }$ be the set of primary and secondary hypotheses, respectively, such that $I=I_{1}^{\prime }\cup I_{2}^{\prime }$.
2. Test the primary (secondary) hypotheses in $I_{1}^{\prime }\;\left(I_{2}^{\prime }\right)$ using the constrained MCP‐Mod test at the level of the sum of their local significance levels, respectively.
3. Reject $H_{I}$ if the test in step 2 is significant for the primary or the secondary hypotheses.

The above test controls the Type I error at level α for each intersection hypothesis because the sum of local significance level is no more than α because of the Bonferroni split. Thus the resulting closed test procedure controls the FWER strongly. In addition, one can construct other graphs to incorporate the primary and the secondary endpoint. For example, change the weight from 1 to x of the edge from $H_{1i}$ to $H_{2i}$ and the weight from 0 to $\left(1-x\right)/2$ of the edges from $H_{1i}$ to $H_{1j}$, where 0≤x≤1 and 1≤i≠j≤3. The resulting graph is also symmetric with respect to the three dose‐control comparisons. Thus the proposed closed MCP‐Mod can also be carried out in a similar fashion. More discussions on symmetric graphs are provided by [44].

## 8 Discussion

In this paper, we show how the closed testing principle can be applied together with MCP‐Mod to define valid level α tests for each intersection test so that in addition to concluding there is a positive dose–response relationship also individual dose‐control comparison can be made. The main advantage is that by using the proposed closed MCP‐Mod is that the additional testing of individual doses against a common control comes for free in terms of multiplicity. When using the optimal (unconstrained) coefficients for the contrast testing, we have a proof that when testing k=2 doses the Type I error rate is controlled. In simulation studies for k>2 we have not observed an inflation of the Type I error rate for the many‐one comparisons, but a formal proof is missing. We have shown which additional assumptions are needed to control the FWER in the strong sense. If one is not comfortable with such additional assumptions, we have proposed the constrained MCP‐Mod as an alternative for the originally proposed optimal contrast tests. When using the constrained MCP‐Mod, no additional assumptions are needed for strict control of the FWER. By the means of simulations studies, we showed that the closed MCP‐Mod with constraints has equal or higher power than the unconstrained version. Furthermore, if the true dose–response is covered by one of the models used in the candidate set, both closed MCP‐Mod version outperform traditional MCP methods such as step‐down Dunnett or Hochberg test. However, in situations where the true dose–response shape is not sufficiently covered by any model in the candidate set, Hochberg and Dunnett are more robust. Concluding, the closed MCP‐Mod approach is designed for confirmatory Phase 3 trials that include more than two doses. In these cases, a definite dose–response relationship presumably has not been established in previous trials but remains of great interest, in addition to the pairwise dose‐control comparisons. Compared to simpler multiple testing procedures, the closed MCP‐Mod approach offers the following advantages: (i) hierarchical testing procedures are prone to lack of power if monotonicity does not hold so that clinical teamsare often reluctant to use sequential testing approaches, and (ii) the Hochberg or step‐down Dunnett procedures are less powerful under the same assumptions as the closed MCP‐Mod approach (non‐monotone efficacy and safety) as long as the “right” candidate model set is used. For its applicability in phase III clinical trials we show how the closed MCP‐Mod can be used testing both primary and secondary endpoints in a confirmatory way.

The closed MCP‐Mod is not a consonant test which can cause some loss of power in specific situations compared to consonant tests such as the step‐down Dunnett test. Part of future research is a consonant version of the closed MCP‐Mod which would require a modification of the weights for each intersection. If we would not recalculate the optimal contrasts for every intersection hypothesis, but rather keep the contrast vector from the global null (all doses) and just rescale it to the doses still present in the intersection, then with the constrained weights, we may get a consonant procedure. It would be interesting to investigate to whether the values of the test statistics increases when eliminating the “weakest” dose (j, say) from an intersection hypthesis and redistributing its weight onto the remaining doses. Since the correlation matrix is a submatrix of the old correlation matrix, we know that the critical value will go down. Thus, if $H_{I}$ was rejected, then we have found that $H_{I-j}$ also gets rejected which is good enough for consonance. Care have to been taken if we re‐calculate the “scalar” variance estimate $\sigma^{2}$ as this may lead to a larger value because the weakest dose also has a small variance. This problem may be alleviated if using the variance estimate from all doses. This whole reasoning does not work anymore if the unconstrained weights are used.

The choice of candidate set is critical and as the correlations between models are usually high, not much penality in terms of multiplicity has to be paid. If there is high uncertainty and quite different models are used in the initial candidate set (causing larger multiplicty adjustments), then an adaptive two‐stage design could be performed and selecting the more relevant models in an interim analysis based on already accumulated data. However, then for the final analysis the data before and after the interima analysis cannot be simply pooled for the joined analysis, but adaptive methods such as combination tests or the conditional error function have to be applied within the proposed closed testing [46]. Another use of an adaptive design stems from the observation that if the total sample size of a trial is fixed, it is usually better to have more doses to adequately describe the dose–response relationship, which comes with a substantial power loss for the individual dose‐control comparisons. One option is therefore to perform an interim analysis to select early on the most promising dose and power the second stage of the trial for the comparison of the selected dose against control.

If one is not interested in performing individual dose‐control comparisons in the framework of MCP‐Mod, but wants to maximize the number of potential model candidates, then the closed testing principle could be used in a different way. Then an individual hypothesis will be linked with a single model and the closed test applied accordingly. Such a strategy will be more relevant for exploratory trials in phase II, where the modelling part to understand the potential dose–response relationship is the main objective.

To summarize, the intended use of the closed MCP‐Mod approach is in Phase 3 clinical trials with more than two doses, where the main objective is to demonstrate efficacy for an individual dose. This is not to be misunderstood, however, as an encouragement to skip Phase 2 dose finding trial. Whenever possible, separate Phase 2 dose finding programs with sufficient dose levels should be performed, using, for example, the original MCP‐Mod approach. In such settings, the subsequent Phase 3 programs will rely on 1, or at most 2, doses and the closed MCP‐Mod approach is not needed. However, we sometimes experienced Phase 3 trials that have been initiated in the absence of a definite dose–response relationship for various reasons. In these settings, establishing a dose–response relationship is of interest, in addition to the pairwise dose‐control comparisons, and the closed MCP‐Mod becomes a viable approach.

## Conflicts of Interest

The authors declare no conflicts of interest.

## Data Availability

Data sharing is not applicable to this article as no real data were analyzed in this study.

## Appendix A

### Distribution of Original MCP‐Mod Test Statistic

The test statistic $T_{m}$ to test $H_{0m}$ of the *m*th model shape is given in Equation (2) in Section 3.2. The final test statistic $T_{\max}=\max_{m}\;T_{m}$ to establish any dose–response relationship is then the maximum of all single contrast tests. Its distribution can be derived from the joint distribution of $\;T={\left(T_{1},\ldots ,T_{M}\right)}^{\prime }$. Under the null hypothesis of no dose–response effect (i.e., $\mu_{0}=\ldots =\mu_{k}$) and the distributional assumptions stated in (1), $T_{1},\ldots ,T_{M}$ jointly follow a central multivariate t distribution with N−k degrees of freedom and correlation matrix $\;R=\left(\rho_{\mathrm{ij}}\right)$, where

(A1)
$$\rho_{\mathrm{ij}}=\frac{\sum_{l=1}^{k}c_{\mathrm{il}}c_{\mathrm{jl}}/n_{l}}{\sqrt{\sum_{l=1}^{k}c_{\mathrm{il}}^{2}/n_{l}\sum_{l=1}^{k}c_{\mathrm{jl}}^{2}/n_{l}}}$$

### Distribution of Closed MCP‐Mod Test Statistic

The final test statistic $T_{\max,I}=\max_{m}T_{m,I}$ for $H_{I}$ is the maximum of all single contrast tests $T_{m,I}$. Its distribution can be derived from the joint distribution of $\;T={\left(T_{1,I},\ldots ,T_{M,I}\right)}^{\prime }$. The distribution is derived similar as given in Distribution of original MCP‐Mod test statistic for the original MCP‐Mod, but modifying the modified correlation $\rho_{\mathrm{ij}}$ given in Equation (A1) to incorporate the intersection specific contrast coefficients $c_{\mathrm{il},I}$. Under the null hypothesis $H_{I}$ of no dose–response effect and the distributional assumptions given in (1), $T_{1,I},\ldots ,T_{M,I}$ jointly follow a central multivariate t distribution with N−k−1 degrees of freedom from the pooled variance estimate in (3) and correlation matrix $\;R_{I}=\left(\rho_{\mathrm{ij},I}\right)$, where

(A2)
$$\rho_{\mathrm{ij},I}=\frac{\sum_{l=0}^{k}c_{\mathrm{il},I}c_{\mathrm{jl},I}/n_{l}}{\sqrt{\sum_{l=0}^{k}c_{\mathrm{il},I}^{2}/n_{l}\sum_{l=0}^{k}c_{\mathrm{jl},I}^{2}/n_{l}}}$$

### Test of Two Doses Against a Comparator With a Global Test Using Optimal Weights

#### Setup

Assume that two doses of a drug are compared with a control treatment where responses to treatment are $N\left(\mu_{i},\sigma^{2}\right)$‐distributed with i=0 indicating control and i=1,2 the two doses. Our general test strategy is as follows:

1. Test $H_{0}:\mu_{1}=\mu_{2}=\mu_{0}\;\text{versus}\;A_{0}:\;\text{not}\;H_{0}$ at level α.
2. If and only if the test from 1. is significant, test both $H_{i}:\mu_{i}\leq \mu_{0}$ versus $A_{i}:\mu_{i}>\mu_{0}$.

We would conclude that there is sufficient evidence for $\mu_{i}>\mu_{0}$ if both $H_{0}$ and $H_{i}$ are rejected.

Notice that 1. is a two‐sided test. Therefore, we cannot generally argue that this strategy keeps the Type I error by the closed test principle.

Regarding the test of $H_{0}$, we now assume that we have an idea of the dose–response shape and are therefore able to calculate optimal contrasts, that is, weights $c=\left(c_{1},c_{2}\right)$ such for the assumed profile $\left(\mu_{0},\mu_{1},\mu_{2}\right)$, the probability of rejecting $H_{0}$ is maximized [9, 33]. More precisely, let

$$\bar{x}=\left(\begin{array}{c} {\bar{x}}_{0}\\ {\bar{x}}_{1}\\ {\bar{x}}_{2} \end{array}\right)∼N\left(\left(\begin{array}{c} \mu_{0}\\ \mu_{1}\\ \mu_{2} \end{array}\right),\sigma^{2}I_{3}\right)$$

be the vector of observed mean responses in the three treatment groups (assuming equal sample sizes for simplicity of notation),

$$\left(\begin{array}{l} k_{1}^{\prime }\\ k_{2}^{\prime } \end{array}\right)=K=\left(\begin{array}{ccc} -\;1 & 1 & 0\\ -\;1 & 0 & 1 \end{array}\right)$$

the contrast matrix such that $K\bar{x}∼N\left(\left(\begin{array}{l} \delta_{1}\\ \delta_{2} \end{array}\right),\sigma^{2}KK^{\prime }\right)$ and $KK^{\prime }=\left(\begin{array}{cc} 2 & 1\\ 1 & 2 \end{array}\right)$.

Then the test for $H_{0}$ is

$$t_{0}=\frac{c^{\prime }K\bar{x}}{\sigma \sqrt{c^{\prime }KK^{\prime }c}}\begin{array}{c} \geq \\ < \end{array}c_{\alpha }$$

where $c_{\alpha }=\Phi^{-1}\left(1-\alpha \right)$ is the 1−α‐quantile of $N\left(0,1\right)$. If $t_{0}\geq c_{\alpha }$, we test $H_{i}:\delta_{i}>0$ with

$$t_{i}=\frac{k_{i}^{\prime }\bar{x}}{\sigma \sqrt{2}}\begin{array}{c} \geq \\ < \end{array}c_{\alpha }.$$

The probability of rejecting either $H_{1}$ or $H_{2}$ is thus given by

(A3)
$$p_{\mathrm{rej}}=P\left(t_{0}\geq c_{\alpha },t_{1}\geq c_{\alpha }\right)+P\left(t_{0}\geq c_{\alpha },t_{1}<c_{\alpha },t_{2}\geq c_{\alpha }\right)$$

The joint distribution of $\left(t_{0},t_{1},t_{2}\right)$ is

(A4)
$$N\left(\left(\begin{array}{c} \frac{c^{\prime }\delta }{\sigma \sqrt{c^{\prime }KK^{\prime }c}}\\ \frac{\delta_{1}}{\sigma \sqrt{2}}\\ \frac{\delta_{2}}{\sigma \sqrt{2}} \end{array}\right),R\right)$$

where

$$R=\left(\begin{array}{cc} 1 & \frac{c^{\prime }KK^{\prime }}{\sigma \sqrt{c^{\prime }KK^{\prime }c}}\\ \frac{KK^{\prime }c}{\sigma \sqrt{c^{\prime }KK^{\prime }c}} & \begin{array}{cc} 1 & \frac{1}{2}\\ \frac{1}{2} & 1 \end{array} \end{array}\right)$$

Note that R has rank 2, that is, the joint distribution of $\left(t_{0},t_{1},t_{2}\right)$ is in a two‐dimensional subspace of $R^{3}$.

Using this joint distribution, we rewrite (A3) as

(A5)
$$p_{\mathrm{rej}}=P\left(t_{0}^{*}\geq c_{\alpha }-\frac{c^{\prime }\delta }{\sigma \sqrt{c^{\prime }KK^{\prime }c}},t_{1}^{*}\geq c_{\alpha }-\frac{\delta_{1}}{\sigma \sqrt{2}}\right)+$$

$$P\left(t_{0}^{*}\geq c_{\alpha }-\frac{c^{\prime }\delta }{\sigma \sqrt{c^{\prime }KK^{\prime }c}},t_{1}^{*}<c_{\alpha }-\frac{\delta_{1}}{\sigma \sqrt{2}},t_{2}^{*}\geq c_{\alpha }-\frac{\delta_{2}}{\sigma \sqrt{2}}\right)$$

where $\left(t_{0}^{*},t_{1}^{*},t_{2}^{*}\right)$ has the distribution $N\left(0,R\right)$.

Obviously, a Type I error inflation cannot occur if one of the hypotheses is wrong, as both *H*_*i*_'s considered in isolation are tested at level α. We can thus restrict investigations to the case δ≤0 and $\delta_{2}\leq 0$. Furthermore, it is very easy to see that if both $c_{1}\geq 0$ and $c_{2}\geq 0$, we have $p_{\mathrm{rej}}\leq P\left(t_{0}^{*}\leq c_{\alpha }\right)=\alpha$ such that the procedure keeps the Type I error by the closed test principle. However, the optimal contrasts can be such that this conditions only holds for either $c_{1}$ or $c_{2}$, not for both. (Note that the case that both $c_{1}$ and $c_{2}$ are smaller than zero will not be considered, as the assumption of a generally decreasing profile when in reality an increase is desired will never be used as the basis for an optimal contrast calculation.) Hence, we will now assume wlog that $c_{1}<0$ and $c_{2}\geq 0$ and show that the setup still keeps the FWER, that is, that $p_{\mathrm{rej}}\leq \alpha$ under this assumption as well (if α≤0.5).

#### Proof

As $p_{\mathrm{rej}}\leq P\left(t_{0}^{*}\geq c_{\alpha }-\frac{c^{\prime }\delta }{\sigma \sqrt{c^{\prime }KK^{\prime }c}},t_{1}^{*}\geq c_{\alpha }-\frac{\delta_{1}}{\sigma \sqrt{2}}\right)+P(t_{0}^{*}\geq c_{\alpha }-\frac{c^{\prime }\delta }{\sigma \sqrt{c^{\prime }KK^{\prime }c}},t_{1}^{*}<c_{\alpha }-\frac{\delta_{1}}{\sigma \sqrt{2}},t_{2}^{*}\geq c_{\alpha })$ for $\delta_{2}\leq 0$, and as c is determined only up to a multiplicative constant, it suffices to consider the case $\delta_{2}=0,c_{2}=1$ and $\sigma^{2}=1$. Hence, $c^{\prime }\delta =c_{1}\delta_{1}$, $\sqrt{c^{\prime }KK^{\prime }c}=\sqrt{2}\sqrt{c_{1}^{2}+c_{1}+1}$ such that (A5) can be written as

(A6)
$$p_{\mathrm{rej}}=P\left(t_{0}^{*}\geq c_{\alpha }-a\delta_{1},t_{2}^{*}\geq c_{\alpha }\right)+P\left(t_{0}^{*}\geq c_{\alpha }-a\delta_{1},t_{1}^{*}\geq c_{\alpha }-\frac{\delta_{1}}{\sigma \sqrt{2}},t_{2}^{*}<c_{\alpha }\right)$$

with $a=\frac{c_{1}}{\sqrt{2}\sqrt{c_{1}^{2}+c_{1}+1}}\leq 0$.

If $c_{1}=0$, then $p_{\mathrm{rej}}<\alpha$ is obvious. Hence, we can assume a<0.

$P\left(t_{0}^{*}\geq c_{\alpha }-a\delta_{1},t_{2}^{*}\geq c_{\alpha }\right)$ is a decreasing function in $\delta_{1}$ and hence achieves its maximum as $\delta_{1}\to -\infty$. For $\delta_{1}\to -\infty$, we have

$$P\left(t_{0}^{*}\geq c_{\alpha }-a\delta_{1},t_{2}^{*}\geq c_{\alpha }\right)\overset{\delta_{1}\to -\infty }{\to }P\left(t_{2}^{*}\geq c_{\alpha }\right)=\alpha$$

Thus, we need $P\left(t_{0}^{*}\geq c_{\alpha }-a\delta_{1},t_{1}^{*}\geq c_{\alpha }-\frac{\delta_{1}}{\sigma \sqrt{2}},t_{2}^{*}<c_{\alpha }\right)=0$ in order to keep α in this case. We now show that $P(t_{0}^{*}\geq c_{\alpha }-a\delta_{1},t_{1}^{*}\geq c_{\alpha }-\frac{\delta_{1}}{\sigma \sqrt{2}},t_{2}^{*}<c_{\alpha })=0$ even holds in general for $\delta_{1}\leq 0$:

The conditional distribution of $\left(\begin{array}{l} t_{1}^{*}\\ t_{2}^{*} \end{array}\right)|t_{0}^{*}.$ is

$$N\left(\left(\begin{array}{l} r_{01}t_{0}^{*}\\ r_{02}t_{0}^{*} \end{array}\right),\left(\begin{array}{cc} 1-r_{01}^{2} & \sqrt{1-r_{01}^{2}}\sqrt{1-r_{02}^{2}}\\ \sqrt{1-r_{01}^{2}}\sqrt{1-r_{02}^{2}} & 1-r_{02}^{2} \end{array}\right)\right)$$

where $r_{01}=\frac{2c_{1}+1}{\sqrt{c_{1}^{2}+c_{1}+1}}$ and $r_{02}=\frac{c_{1}+2}{\sqrt{c_{1}^{2}+c_{1}+1}}$. Thus, $\text{corr}\left(t_{1}^{*},t_{2}^{*}|t_{0}^{*}.\right)=1$ in line with the degenerate distribution of $\left(t_{0}^{*},t_{1}^{*},t_{2}^{*}\right)$.

Consequently, we have

$$\frac{t_{1}^{*}-r_{01}t_{0}^{*}}{\sqrt{1-r_{01}^{2}}}=\frac{t_{2}^{*}-r_{02}t_{0}^{*}}{\sqrt{1-r_{02}^{2}}}$$

and thus

$$P\left(t_{1}^{*}\geq c_{\alpha }-\frac{\delta_{1}}{\sqrt{2}},t_{2}^{*}<c_{\alpha }|t_{0}^{*}.\right)=P\left(\frac{c_{\alpha }-\frac{\delta_{1}}{\sqrt{2}}-r_{01}t_{0}^{*}}{\sqrt{1-r_{01}^{2}}}\leq \frac{t_{1}^{*}-r_{01}t_{0}^{*}}{\sqrt{1-r_{01}^{2}}}<\frac{c_{\alpha }-r_{02}t_{0}^{*}}{\sqrt{1-r_{02}^{2}}}|t_{0}^{*}.\right)$$

Obviously this is 0 if

(A7)
$$\frac{c_{\alpha }-\frac{\delta_{1}}{\sqrt{2}}-r_{01}t_{0}^{*}}{\sqrt{1-r_{01}^{2}}}\geq \frac{c_{\alpha }-r_{02}t_{0}^{*}}{\sqrt{1-r_{02}^{2}}}$$

Solving (A7) for $t_{0}^{*}$ leads to

$$t_{0}^{*}\geq \frac{1+c_{1}}{\sqrt{c_{1}^{2}+2c_{1}+1}}c_{\alpha }-a\delta_{1}\geq \frac{1+c_{1}}{\sqrt{{\left(1+c_{1}\right)}^{2}}}c_{\alpha }-a\delta_{1}=c_{\alpha }-a\delta_{1}$$

since $c_{1}\leq 0$ and $\frac{1+c_{1}}{\sqrt{{\left(1+c_{1}\right)}^{2}}}=-1$ for $c_{1}\leq -1$ and 1 otherwise. Hence, for $c_{1}>-1$, $P\left(t_{1}^{*}\geq c_{\alpha }-\frac{\delta_{1}}{\sqrt{2}},t_{2}^{*}<c_{\alpha }|t_{0}^{*}.\right)=0$ holds for all values of $t_{0}^{*}$ and for $c_{1}\leq -1$, it holds as long as $t_{0}^{*}\geq -c_{\alpha }-a\delta_{1}$. Now, as the probability (A6) has $t_{0}^{*}\geq c_{\alpha }-a\delta_{1}$ and as $c_{\alpha }\geq -c_{\alpha }$ for α≤0.5, the claim is proved.

If there is more than a single set c of contrast weights, this consideration holds for each of them. Hence, if we set up several such **c**_*i*_'s and test $H_{0}$ first based on the multivariate normal distribution of several test statistics like $t_{0}$ (Bretz et al., 2006), the proofs extends to this situtation as well.

### Calculation of Optimal Weights With Constraints

Without the constraint, the solution of (6) is identical to the originally suggested optimal contrasts from Bretz et al. [9]. This can be seen from the following consideration:

The problem

(A8)
$$m_{1}=\max\frac{w_{m}^{\prime }K\mu_{m}^{0}}{\sqrt{w_{m}^{\prime }{\mathrm{KA}}_{m}K^{\prime }w_{m}}}$$

and the problem

(A9)
$$m_{2}=\max\frac{c_{m}^{\prime }\mu_{m}^{0}}{\sqrt{c_{m}^{\prime }A_{m}c_{m}}}\;\text{subject}\;\text{to}\;c_{m}^{\prime }1_{k+1}=0$$

yield identical solutions, because on the one hand, setting $c_{m}=K^{\prime }w_{m}$ implies $c_{m}^{\prime }1_{k+1}=0$, as $K1_{k+1}=0$. Hence, $m_{2}\geq m_{1}$. On the other hand, if $c_{m}^{\prime }1_{k+1}=0$, then there must be some $w_{m}\in R^{k}$ such that $c_{m}$ can be written as $c_{m}=K^{\prime }w_{m}$, since the rows of the $k\times \left(k+1\right)$‐matrix K provide a base of the *k*‐dimensional hyperspace orthogonal to $1_{k+1}$ in $R^{k+1}$. Consequently, $m_{2}\leq m_{1}$. It follows that $m_{2}=m_{1}$ and that if $w_{m}$ maximizes (A8), then $K^{\prime }w_{m}$ maximizes (A9). Thus, the suggested modification just adds the additional constraint $w_{\mathrm{mi}}\geq 0$ for all i=1,…,k to the originally suggested contrasts.

Note that (6) without the constraint is a generalized eigenvalue problem with the solution $w_{m}^{*}={\left({\mathrm{KA}}_{m}K^{\prime }\right)}^{-1}K\mu_{m}^{0}$ and the optimal contrast on the original scale can be re‐calculated based on $c_{m}^{*}={K^{\prime }w}_{m}^{*}$. Maximizing (A8) under the additional constraint that $w_{\mathrm{mi}}\geq 0$ for all i=1,…,k, is equivalent to minimizing $w_{m}^{\prime }{\mathrm{KA}}_{m}K^{\prime }w_{m}$ under the constraints $w_{m}^{\prime }K\mu_{m}^{0}=\kappa$ (with arbitrary κ>0) and $w_{\mathrm{mi}}\geq 0$ for all i=1,…,k. This is a quadratic programming problem and can be solved with standard software, such as the quadprog R‐package [47]. The resulting optimal weights are defined up to an arbitrary positive proportionality constant.
